# Adequate Fruit and Vegetable Consumption Is Associated with Protection Against Cognitive Impairment No Dementia (CIND): Findings from the ELSI Cross-Sectional Population Study

**DOI:** 10.3390/nu18030496

**Published:** 2026-02-02

**Authors:** Amanda Maria de Sousa Romeiro, Gilberto Sousa Alves, Cesar de Oliveira, Erika Aparecida Silveira

**Affiliations:** 1 Postgraduate Program in Health Sciences, Faculty of Medicine, Federal University of Goiás, Goiânia 74690-900, Brazil; 2 Department of Medicine I, Federal University of Maranhão, São Luís 65080-805, Brazil; gsalves123@hotmail.com; 3 Department of Epidemiology & Public Health, Institute of Epidemiology & Health Care, University College London, London WC1E 6BT, UK; c.oliveira@ucl.ac.uk

**Keywords:** dementia, fruits, healthy diet, nutrition, older people, vegetables

## Abstract

**Background:** Dementia is a growing global public health concern and identifying modifiable risk and protective factors is crucial for its prevention. Fruits and vegetables, due to their antioxidant and anti-inflammatory properties, may offer neuroprotective benefits. This study aims to investigate the prevalence of adequate fruit and vegetable consumption and its association with dementia and cognitive impairment no dementia (CIND) in individuals aged 50 years and older. **Methods:** This cross-sectional, population-based study analysed data from 2865 participants in the second wave (2019–2021) of the Brazilian Longitudinal Study of Ageing (ELSI-Brazil). CIND was defined as a global cognitive Z-score ≤ −1.5, and dementia as cognitive decline with impairment in at least one instrumental activity of daily living. Adequate consumption of fruits, vegetables, and both combined (FV) was defined as daily intake on all seven days of the week. Associations were assessed using multivariate Poisson regression models, with prevalence ratios (PRs) and 95% confidence intervals (CIs). **Results:** The study sample consisted of 2865 participants. The prevalence of adequate fruit consumption was 58.08% (95% CI: 56.3–59.9), vegetables 44.14% (95% CI: 42.31–45.9), and FV 32.18% (95% CI: 30.5–33.9). Adequate vegetable consumption was significantly associated with CIND (PR: 0.19; 95% CI: 0.07–0.50; *p* < 0.001), while adequate fruit consumption was associated with higher prevalence of CIND (PR: 1.47; 95% CI: 1.22–1.77) and FV (PR: 0.20; 95% CI: 0.07–1.58; *p* = 0.003). No significant association was observed between fruit, vegetable, and FV consumption and dementia. **Conclusions:** Adequate vegetable and combined FV consumption were protective against CIND, though not associated with dementia. Nonetheless, overall adequate intake remains low in older Brazilian adults.

## 1. Introduction

The ageing process is a global and complex issue in public policy related to geriatrics, driven by an increase in age-related diseases such as neurodegenerative conditions, notably dementias [1,2,3]. The decline of cognitive functions characterises cognitive impairment, which can progress into various types of dementia [4,5]. Currently, around 55 million people are affected by dementia, and projections suggest that by 2050, this number could rise to 139 million cases [6].

Several factors are associated with an increased risk of dementia, including diabetes, hypertension, low education levels, obesity, depression, physical inactivity, smoking, alcohol abuse, traumatic brain injury, air pollution, dietary habits, and low social participation [7,8,9,10]. However, food consumption variables are less studied but hold significant potential as both risk and protective factors for dementia, particularly fruits and vegetables, because of their rich content of vitamins, minerals, and polyphenols that may help prevent dementia [10,11,12,13].

Vitamins and polyphenols found in fruits and vegetables play a crucial role in reducing neurotoxicity and mitigating free radical damage, thereby reducing oxidative stress. These compounds act as natural anti-inflammatory and antithrombotic agents, in addition to improving cerebral perfusion. These effects contribute to improved cognitive function, including memory, locomotor speed, sensory processing, and information processing [14,15,16].

There are a few studies worldwide exploring the association of fruit and vegetable consumption with dementia and cognitive impairment no dementia (CIND) in older adults [17,18,19,20,21]. In Brazil, only one study examined the possible association with cognitive performance [22]. Given the biological plausibility of the neuroprotective effects of fruits and vegetables and the scarcity of evidence in South America, we hypothesised that adequate daily consumption would act as a protective factor against dementia and cognitive impairment no dementia (CIND). Therefore, the objectives of this research were: (I) to describe the prevalence of adequate fruit and vegetable consumption; (II) to investigate their association with dementia and CIND in individuals aged 50 years and older.

## 2. Material and Methods

### 2.1. Study Design and Participants

This study employed a cross-sectional design using data from a nationally representative survey on the ageing of the Brazilian population aged 50 years and over, the Brazilian Longitudinal Study of Ageing (ELSI-Brazil) [23]. Data from the second wave (2019–2021) were analysed, comprising 9949 participants residing in rural and urban areas across the five regions of Brazil. Data collection was carried out through individual home interviews, using questionnaires that addressed sociodemographic characteristics, lifestyle factors, physical and mental health, as well as physical tests and measurements [24].

Individuals aged 50 years and older were selected, including those who responded directly or whose answers were provided by a family member or close friend. Participants who reported having or using medications for Parkinson’s disease or severe psychiatric disorders were excluded, as well as those who answered “I do not know” or did not respond to questions related to cognitive function.

### 2.2. Sociodemographic Variables

The sociodemographic variables analysed included sex, age, self-reported race/skin colour, income, and education. Sex was categorised as female or male. Age was stratified into three groups: 50–64 years, 65–74 years, and 75 years or older. Self-declared skin colour was classified according to the Brazilian Institute of Geography and Statistics as White, Brown, and Black/Others, with “Others” being individuals who identified as Asian or Indigenous due to the small sample size. Individual income was divided into quartiles of 25%, using the detailed command variable for classification. Education was categorised into three groups: illiterate (no formal education), up to elementary school (up to seven years of study), and high school or higher education (eight or more years of study).

### 2.3. Lifestyle Variables

Lifestyle variables included living with a partner (yes or no), smoking (never smoker and former/current smoker), and excessive alcohol consumption (weekly intake of ≥14 standard drinks or four standard drinks per day for men, and ≥7 standard drinks per week or three standard drinks per day for women) [25]. Sedentary behaviour was defined as an inactive level of physical activity, involving individuals who engaged in walking and moderate/vigorous exercise for less than 150 min per week [26].

### 2.4. Morbidities

Obesity was defined according to Silveira et al. (2020) [27], who evaluated the accuracy of BMI cut-off points for older adults in Brazil, a population comparable to this study. In this model, obesity was defined as BMI ≥ 25 kg/m^2^ for men and BMI ≥ 26.6 kg/m^2^ for women [27]. Systemic arterial hypertension was diagnosed based on systolic and/or diastolic blood pressure measurements ≥ 140/90 mmHg [28]. Depressive symptoms were assessed using the 8-item Centre for Epidemiologic Studies Depression Scale (CESD-8), which assigns scores between 0 and 8. Scores ≥ 4 were considered indicative of depressive symptoms [29,30]. Chronic conditions, including diabetes mellitus (DM), cardiovascular disease, and cerebrovascular disease, were identified through self-report, considering prior medical diagnosis and/or the use of specific medications for these morbidities [24].

### 2.5. Outcomes: CIND and Dementias

The assessment of cognitive function was divided into different questions covering five cognitive subdomains, each with varying scores. Temporal orientation was assessed by the correct recognition of the day, month, year, and day of the week, with scores ranging from 0 to 4 points. Semantic verbal fluency was measured by the number of animals mentioned by the participant within one minute, with scores ranging from 0 to 20 points. Episodic memory was assessed through the “10-word list test” for immediate and delayed recall, with scores ranging from 0 to 20 points. Prospective memory was analysed based on the participant’s ability to remember to write his/her name at the end of the test, as previously instructed by the interviewer, with scores ranging from 0 to 4 points. Semantic memory was assessed through questions related to general and political knowledge, with scores ranging from 0 to 4 points [31].

Global cognitive function was assessed in two stages. First, for each cognitive subdomain, the scores were standardized by calculating the Z-score using the formula: Z-score = (individual score − subdomain mean) divided by the subdomain standard deviation. Then, the arithmetic mean of these standardized Z-scores was obtained across all assessed subdomains. From this final mean, the overall cognitive Z-score was calculated, again applying the standardization formula described above [31,32,33]. For individuals unable to complete the questionnaire of cognitive domains, a close friend or family member completed the 16-item Brazilian version of the Informant Questionnaire on Cognitive Decline in the Elderly (IQCODE). A Likert scale from 1 to 5 was used to evaluate each question, considering the changes observed in the participant over the past two years [34]. 

CIND was defined as a global cognitive Z score of ≤−1.5 standard deviations. The diagnosis of dementia was established based on two criteria: (1) a global cognitive Z score of <−1.5 standard deviations accompanied by impairment in one or more instrumental activities of daily living (IADLs), including financial management, use of transportation, telephone/cell phone, and medication administration; or (2) IQCODE score of ≥3.4 [31].

### 2.6. Fruits and Vegetables Consumption

The adequate frequency of fruit and vegetable consumption was defined as daily consumption over the seven days of the week [35,36]. Fruit consumption was assessed through a self-reported questionnaire on fruits and natural juices, with the questions: “How many days of the week do you usually consume fruit?” and “How many days of the week do you usually consume natural fruit juice?”. Vegetable consumption was measured by the question: “How many days of the week do you usually consume vegetables (such as cabbage, carrots, chayote, eggplant, zucchini, lettuce, tomatoes)?”. For both, adequate consumption corresponded to 7 points. The sum of the answers to these questions, totalling 14 points, was considered indicative of adequate consumption of fruits and vegetables combined (FV).

### 2.7. Statistical Analysis

Bivariate analysis was conducted to evaluate the prevalence and association of fruit and vegetable consumption and FV with sociodemographic variables (sex, age group, skin colour, income, education, and living with a partner), lifestyles (smoking, excessive alcohol consumption and sedentary behaviour), and morbidities (obesity, hypertension, diabetes mellitus, depression, cardiovascular diseases, and cerebrovascular diseases), as well as the two primary outcomes (CIND and dementia). These variables were selected because they represent well-established modifiable risk factors for dementia reported in the literature [10]. The prevalence ratio (PR) was calculated using Poisson regression, with a 95% confidence interval (95% CI). To determine the PR of morbidity variables, the ‘no’ category served as the reference. Conversely, for the other sociodemographic variables and lifestyles, the category representing the best scenario was used as the reference.

For the multivariate analysis, two models were conducted to examine the association between adequate fruit, vegetable, and FV consumption and the outcomes of CIND and dementia. Associations were considered statistically significant if *p* < 0.05. Variables with a *p*-value less than 0.20 in the bivariate analysis were included [37] in the multivariate analysis at the following levels: Model 1, adjusted for sociodemographic variables, and Model 2, adjusted for Model 1 plus lifestyle and morbidity factors. Before conducting the analyses, the database was calibrated using the svyset command according to the sampling structure and recommendations provided by the ELSI-Brazil study documentation, incorporating information on primary sampling units (PSUs), strata, and survey weights. Considering the complex sampling design, all analyses were then performed with the svy prefix in Stata/MP 12.1 to ensure population-representative estimates.

## 3. Results

**Figure 1:**
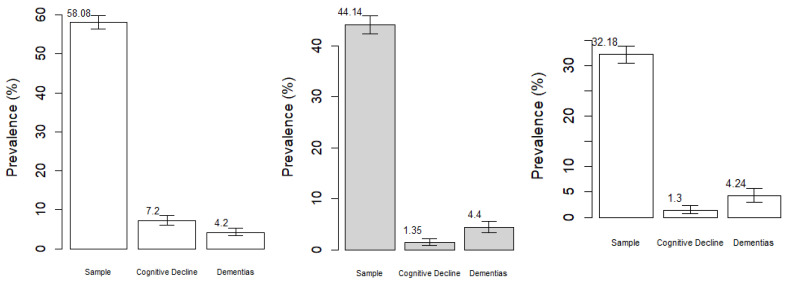
Prevalence of adequate consumption of fruits, vegetables, and FV in the sample and according to the occurrence of dementias and cognitive impairment no dementia (CIND) in adults aged 50 years or older in Brazil (*n* = 2865).

After applying the eligibility criteria, the sample included 2865 participants. The prevalence of adequate fruit consumption was 58.08% (95% CI: 56.3–59.9), with 7.2% (95% CI: 6.01–8.6) classified as CIND and 4.2% (95% CI: 3.34–5.35) as having dementia. Adequate vegetable consumption showed a prevalence of 44.14% (95% CI: 42.31–45.9), with 1.35% (95% CI: 0.8–2.1) presenting CIND and 4.2% (95% CI: 3.34–5.35) dementia. For combined adequate fruit and vegetable consumption, the prevalence was 32.18% (95% CI: 30.5–33.9), with 1.3% (95% CI: 0.67–2.3) exhibiting CIND and 4.24% (95% CI: 3.03–5.75) dementia (Figure 1).

In the bivariate analysis, adequate fruit consumption was associated with sex, skin colour, and education (*p* < 0.05). Age group, smoking, obesity, and diabetes mellitus were added to the multivariate model. Adequate vegetable consumption was associate with skin colour, education, and diabetes mellitus, with sex, depression, cardiovascular disease, and cerebrovascular disease included in the multivariate analysis. Adequate FV consumption was associated with sex, education, and diabetes mellitus, and the multivariate model additionally considered age group, skin colour, per capita income, sedentary lifestyle, obesity, and cerebrovascular disease (Table 1).

CIND was associated with inadequate FV consumption (*p* < 0.001) and adequate fruit consumption (*p* = 0.014). When stratifying by frequency, CIND was associated with vegetable consumption on 0 to 4 days/week and 5 to 6 days/week (*p* < 0.001), and with fruit consumption on 5 to 6 days/week and 7 days/week (*p* = 0.004). No statistically significant associations were observed between dementias and adequate consumption of fruits and FV (Table 2).

In the multivariate analysis, adequate fruit consumption was a risk factor for CIND in Model 1 adjusted for sociodemographic variables and in Model 2 adjusted for lifestyle and morbidity variables (Table 3). Adequate fruit consumption was also associated with individuals aged 65 to 74 years and those with higher educational levels. Adequate vegetable consumption, in contrast, was a protective against CIND in both models and was associated with older age, white and black/other skin colour, secondary/higher education, hypertension, and depression. Adequate FV consumption was inversely associated with CIND in Model 1 (PR: 0.20; 95% CI: 0.07–0.057; *p* = 0.003) and Model 2 (PR: 0.20; 95% CI: 0.07–1.58; *p* = 0.003) and was related to individuals aged 50–64 and 65–75 years, those in the second income quartile, with elementary and secondary/higher education, diabetes mellitus and cerebrovascular disease (Table 3).

After adjustments for sociodemographic, lifestyle, and morbidity variables, dementia was not associated with adequate fruit, vegetables or FV consumption (Table 4). In Model 1, adequate fruit consumption was associated with being female, aged 65 and 74 years, and having elementary or secondary/higher education. Adequate vegetable consumption was associated with individuals aged 65 to 74 and 75 or older, black/other skin colour, and elementary or secondary/higher education. In Model 2, it was additionally associated with hypertension and diabetes mellitus. Adequate FV consumption was associated with being female, aged 50 to 64 and 65 to 74 years, elementary or secondary/higher education, obesity and cerebrovascular disease (Table 4).

## 4. Discussion

The current study investigates the relationship between fruit consumption and the incidence of age-related cognitive decline in a representative sample of the Brazilian population. Our findings highlight an association between lower vegetable consumption and a diagnosis of CIND. Conversely, daily fruit consumption was linked to CIND. Other variables, such as gender, education, and skin colour, were also related to adequate FV consumption. Finally, the occurrence of dementia was not associated with FV consumption. Our data provides important insights into the connection between dietary habits and the development of age-related cognitive decline.

The 32.18% prevalence of adequate FV consumption in this sample differs from the main nationally representative studies on the topic, which reported a 12.9% prevalence of appropriate consumption of these foods [38]. In low- and middle-income countries, the study using the first wave of the WHO Study of Global Ageing (SAGE) found prevalence rates of adequate fruit and vegetable consumption of 9.1% in China, 5.4% in Ghana, 3.3% in India, 9.7% in Mexico, 17.9% in Russia, and 7.8% in South Africa among individuals over 60 years old [39]. Currently, the WHO recommends a minimum consumption of 400 g/day of fruits and vegetables, mainly for preventing chronic diseases [40]. The differences between countries highlight the importance of sociocultural factors in shaping the population’s eating habits.

A small number of studies have investigated the association between fruit, vegetable, and FV consumption. The CIND observed in this study aligns with the findings of two studies conducted in older Chinese adults, which reported that vegetable intake [41], and especially the combined consumption of fruits and vegetables [42], decreased the risk of mild cognitive impairment. This highlights the importance of the antioxidant and anti-inflammatory properties of these foods, which inhibit pro-inflammatory cytokines and protect synaptic connections from free radical damage in neuronal tissues [43].

The finding that fruit consumption is a risk factor and vegetable consumption is protective against CIND is intriguing and may result from several potential factors. One possibility is the increased time needed for food preparation. In this case, subclinical changes in functionality would favour the consumption of fruit, which requires no preparation, over adequate vegetable intake, which involves more complex steps such as washing, cooking, and others [44]. Furthermore, increased intake of fruits and fruit juices, which have a high glycaemic index, is linked to greater insulin and glucose spikes, triggering the development of insulin resistance and other mechanisms that ultimately cause increased inflammation, higher oxidative stress, and neuronal signalling dysfunction [45], and decreased the odds of pre-frailty [46].

However, when the exposure variables, in this case fruit and vegetable consumption, are obtained from questionnaires answered by close respondents rather than the participant themselves, the measurement of exposure becomes more susceptible to error. Even when the participant answers themselves, measurement error can still occur due to memory bias, especially among individuals with cognitive decline, who may not accurately recall the amount of food consumed throughout the week. In linear models, such classification errors can result in significant biases, including overestimation or underestimation of the associations between exposure and outcome [47].

No significant association was found between adequate fruit, vegetable, and FV consumption and the occurrence of dementia. Similar results were observed in a study conducted in the Netherlands with older adults, which also found no significant relationship between vegetable consumption and the risk of dementia [48], as well as in a study conducted in the United Kingdom with adults and older adults, which found no consistent association between varied fruit and vegetable consumption and a lower risk of dementia [49]. In contrast, data from the Framingham Heart Study demonstrated that cumulative consumption of flavonoid-rich fruits, starting in middle age, was associated with a 44% reduction in the risk of dementia from all causes [50].

The low prevalence of individuals diagnosed with dementia in this study sample may also, in part, explain the lack of an observed association. This observation highlights the instrumental differences used by the study to measure cognitive domains. Thus, creating harmonized protocols, such as the Harmonized Cognitive Assessment Protocol (HCAP) initiative, could be an advantage for calibrating these measures and the validity of dementia estimates, even contributing to comparison with similar studies from other countries [51]. Despite the limited power, these association estimates can be valuable for future studies, strengthening scientific transparency and avoiding publication bias.

Adequate consumption of fruits, vegetables, and FV was associated with higher education, especially among those with the highest educational attainment. These findings were also reported in a cross-sectional study of fruit and vegetable intake among adults conducted across 21 European countries [52] and in a Brazilian study involving individuals aged 60 and above [53]. People with less education tend to be more influenced by factors like price, ease of access, and satiety, which can lower their likelihood of purchasing nutritionally rich foods such as fruits and vegetables [54].

While younger-old adults have higher overall vegetable intake, the oldest-old are more likely to achieve adequate vegetable consumption than adequate fruit consumption. This distinction is corroborated by another study conducted within the first wave of the same ELSI-Brazil study [22], which demonstrated higher vegetable consumption among older adults, as well as by a Chinese study that reported higher fruit consumption among younger adults [38]. Furthermore, in this study, individuals who self-identified as white consumed adequate amounts of fruits and vegetables compared to those of mixed race, Black, Asian, or Indigenous skin colour. These skin colour-related differences may be influenced by socioeconomic and demographic conditions that limit access to and promotion of these foods. In the Brazilian context, the mixed-race population is particularly vulnerable to these limitations [55].

It was observed that individuals with hypertension, DM, and obesity had adequate consumption of fruits and vegetables, which may serve as a protective factor against CIND and the development of dementia. Although, this finding may reflect a reverse causality bias, where the outcome precedes and results from the exposure, inherent to the cross-sectional design of the study and to outcomes with long preclinical or subclinical periods, since the temporal order between exposure and outcome cannot be established [56]. And this condition may be reflected in individuals with chronic diseases who frequently receive nutritional guidance from health professionals to adopt healthier eating habits, including increasing the intake of these foods as part of the management of their condition.

This study has some limitations, such as: (I) risk of reverse causality; (II) potential for recall bias in dietary assessment; (III) the variable related to fruit and vegetable consumption did not include detailed information on the type, quality, or preparation methods of these foods, which could have limited a more precise analysis of their effects on cognitive outcomes; (IV) the low prevalence of dementia may have reduced the statistical power for detecting a possible association; (V) possible selection bias due to exclusion of participants with missing cognitive assessments.

For future research, it is recommended that longitudinal studies be conducted to monitor individuals over time, thereby addressing the limitations inherent in cross-sectional studies. Additionally, it is recommended that nutritional assessment tools be improved, given the complexity of dietary habits and their potential role as protective factors against CIND. Large-scale studies with nationally representative samples could provide stronger evidence. Successfully increasing fruit and vegetable intake among older adults depends on coordinated national policies that promote consumption and make access affordable.

## 5. Conclusions

The prevalence of sufficient fruit consumption was just over 50%. In contrast, the levels of sufficient vegetable and FV consumption were quite low, indicating the low consumption of these foods among the Brazilian population. The association between sufficient vegetable and FV consumption served as a protective factor against CIND. No connection was found between sufficient fruit and vegetable consumption and dementia. The population’s dietary habits and their influence on cognitive health can help identify risk and protective factors related to CIND and dementia, supporting the development of preventive strategies and encouraging healthy and cognitively active ageing.

## Figures and Tables

**Table 1:** Prevalence and association between adequate consumption of fruits, vegetables, and FV with sociodemographic variables, lifestyle, and morbidities among Brazilians aged 50 years and older (*n* = 2865).

	*n* (%)	Fruits (*n* = 1668)		*p*-Value *	Vegetables (*n* = 1262)		*p*-Value *	FV (*n* = 920)		*p*-Value *
		Prevalence *n* (%)	PR Crude (95% CI)		Prevalence *n* (%)	PR Crude (95% CI)		Prevalence *n* (%)	PR Crude (95% CI)	
Sex				**0.028**			0.067			**<0.001**
Female	1350 (47.12)	832 (61.63)	1.10 (1.01–1.20)		649 (48.15)	1.11 (0.99–1.23)		500 (37.09)	1.26 (1.09–1.44)	
Male	1515 (52.88)	832 (54.92)	1.00		613 (40.57)	1.00		420 (27.80)	1.00	
Age				0.082			0.202			0.078
50 to 64	1582 (55.22)	883 (55.82)	1.00		679 (42.95)	1.00		475 (30.04)	1.00	
65 to 74	844 (29.46)	525 (62.20)	1.10 (1.00–1.19)		388 (46.25)	1.09 (0.99–1.21)		296 (35.28)	1.16 (1.02–1.33)	
75 or more	439 (15.32)	256 (58.31)	1.01 (0.88–1.14)		195 (44.42)	1.08 (0.92–1.26)		149 (33.94)	1.15 (0.95–1.40)	
Skin colour				**0.034**			**0.029**			0.162
White	1534 (53.64)	982 (64.02)	1.19 (0.96–1.48)		715 (46.70)	1.18 (0.95–1.46)		536 (35.01)	1.25 (0.98–1.60)	
Brown	1066 (37.27)	553 (51.88)	0.99 (0.81–1.22)		424 (39.85)	1.00		303 (28.48)	1.00	
Black and others ^∞^	260 (9.09)	128 (49.23)	1.00		121 (46.72)	1.31 (1.07–1.61)		80 (30.89)	1.27 (0.91–1.78)	
Per capita income				0.942			0.667			0.186
1º quartile	698 (24.36)	411 (58.88)	1.00		298 (42.63)	1.00		211 (30.23)	1.00	
2º quartile	815 (28.45)	480 (58.90)	1.01 (0.91–1.12)		368 (45.10)	1.08 (0.94–1.23)		277 (34.03)	1.19 (0.98–1.43)	
3º quartile	644 (22.48)	367 (56.99)	0.99 (0.89–1.11)		279 (43.53)	1.00 (0.87–1.15)		198 (30.94)	1.06 (0.88–1.28)	
4º quartile	708 (24.71)	406 (57.34)	1.02 (0.93–1.12)		317 (44.77)	1.08 (0.92–1.26)		234 (33.10)	1.17 (0.98–1.39)	
Education				**<0.001**			**<0.001**			**<0.001**
Illiterate	271 (9.51)	103 (38.01)	1.00		68 (25.19)	1.00		50 (18.52)	1.00	
Primary	1638 (57.49)	883 (53.91)	1.51 (1.02–2.23)		633 (38.69)	1.63 (1.15–2.32)		415 (25.37)	1.52 (0.94–2.46)	
Secondary/Higher	940 (32.99)	670 (71.28)	1.90 (1.26–2.88)		553 (59.02)	2.40 (1.64–3.50)		448 (47.81)	2.67 (1.63–4.35)	
Living with a partner				0.306			0.327			0.418
No	1238 (43.21)	717 (57.92)	1.00		529 (42.87)	1.00		393 (31.85)	1.00	
Yes	1627 (56.79)	947 (58.21)	1.06 (0.95–1.18)		733 (45.11)	1.08 (0.93–1.25)		527 (32.43)	1.08 (0.90–1.29)	
Smoking				0.064			0.848			0.356
No	1930 (67.53)	1162 (60.21)	1.00		858 (44.57)	0.99 (0.87–1.12)		644 (33.45)	1.08 (0.91–1.28)	
Yes	928 (32.47)	501 (53.99)	1.10 (0.99–1.22)		403 (43.47)	1.00		276 (29.77)	1.00	
Excessive alcohol consumption				0.423			0.918			0.431
No	2354 (95.15)	1345 (57.14)	1.00		1066 (45.40)	1.01 (0.77–1.33)		785 (33.43)	1.17 (0.79–1.73)	
Yes	120 (4.85)	71 (59.17)	1.10 (087–1.37)		54 (45.00)	1.00		35 (29.17)	1.00	
Sedentarism				0.367			0.625			0.089
No	550 (20.31)	319 (58.00)	1.06 (0.93–1.21)		258 (47.08)	1.04 (0.88–1.24)		201 (36.68)	1.20 (0.97–1.49)	
Yes	2158 (79.69)	1258 (58.29)	1.00		925 (42.94)	1.00		663 (30.78)	1.00	
Obesity				0.108			0.059			0.161
No	883 (34.89)	495 (56.06)	1.00		364 (41.22)	1.00		269 (30.46)	1.00	
Yes	1648 (65.11)	983 (59.65)	1.09 (0.98–1.22)		740 (45.01)	1.14 (0.99–1.31)		530 (32.24)	1.13 (0.95–1.35)	
Hypertension				0.967			0.208			0.214
No	1120 (39.19)	653 (58.30)	1.00		482 (43.07)	1.00		345 (30.83)	1.00	
Yes	1738 (60.81)	1009 (58.06)	1.00 (0.91–1.11)		775 (44.72)	1.10 (0.95–1.26)		573 (33.06)	1.12 (0.94–1.34)	
DM				0.192			**0.017**			**0.014**
No	2407 (84.37)	1384 (57.50)	1.00		1030 (42.90)	1.00		748 (31.15)	1.00	
Yes	446 (15.63)	276 (61.88)	1.08 (0.96–1.21)		229 (51.35)	1.15 (1.03–1.30)		171 (38.34)	1.22 (1.04–1.43)	
Depression				0.311			0.093			0.385
No	2062 (79.22)	1232 (59.75)	1.00		947 (46.02)	1.00		689 (33.48)	1.00	
Yes	541 (20.78)	294 (54.34)	0.92 (0.78–1.08)		214 (39.63)	0.87 (0.73–1.02)		156 (28.89)	0.90 (0.71–1.14)	
Heart Disease				0.544			0.135			0.210
No	2686 (93.75)	1556 (57.93)	1.00		1170 (43.62)	1.00		852 (31.77)	1.00	
Yes	179 (6.25)	108 (60.34)	1.05 (0.90–1.21)		92 (51.98)	1.19 (0.95–1.49)		68 (38.42)	1.18 (0.91–1.52)	
Cerebrovascular Diseases				0.903			0.057			0.095
No	2757 (96.30)	1599 (58.00)	1.00		1205 (43.79)	1.00		877 (31.87)	1.00	
Yes	106 (3.70)	650 (61.32)	0.99 (0.80–1.22)		57 (54.29)	1.25 (0.99–1.56)		43 (40.95)	1.26 (0.96–1.66)	

PR: prevalence ratio, 95% CI: 95% confidence interval; DM: diabetes mellitus; * F Test; ^∞^ skin colour variable: black (*n* = 271) + yellow/indigenous (*n* = 19), *p*-values in bold indicate statistical significance.

**Table 2:** Prevalence and association between the frequencies of consuming fruits, vegetables, and FV with dementia and cognitive impairment no dementia (CIND) in Brazilians aged 50 years or older (*n* = 2865).

	*n* (%)	CIND (*n* = 154)		*p*-Value *	Dementias (*n* = 151)		*p*-Value *
		Prevalence *n* (%)	PR Crude (95% CI)		Prevalence *n* (%)	PR Crude (95% CI)	
Fruits				**0.014**			0.082
Inadequate	1201 (41.92)	34 (2.83)	1.00		80 (6.66)	1.61 (0.94–2.78)	
Adequate	1664 (58.08)	120 (7.21)	2.45 (1.20–5.02)		71 (4.27)	1.00	
Vegetables				**<0.001**			0.109
Inadequate	1597 (55.86)	137 (8.58)	9.70 (3.51–26.80)		95 (5.95)	1.46 (0.92–2.33)	
Adequate	1262 (44.14)	17 (1.35)	1.00		55 (4.36)	1.00	
Fruits and Vegetables				**<0.001**			0.155
Inadequate	1939 (67.82)	142 (7.32)	7.81 (2.63–23.21)		111 (5.72)	1.43 (0.88–2.35)	
Adequate	920 (32.18)	12 (1.30)	1.00		39 (4.24)	1.00	
Fruits (day/week)				**0.004**			0.174
0 to 4	870 (30.37)	18 (2.07)	1.00		62 (7.13)	1.61 (0.98–2.65)	
5 to 6	331 (11.55)	16 (4.83)	3.11 (1.55–6.26)		18 (5.44)	1.63 (0.68–3.91)	
7	1664 (58.08)	120 (7.21)	3.93 (1.57–9.87)		71 (4.27)	1.00	
Vegetables (day/week)				**<0.001**			0.257
0 to 4	1160 (40.57)	123 (10.60)	11.95 (4.21–33.94)		69 (5.95)	1.39 (0.86–2.25)	
5 to 6	437 (15.29)	14 (3.20)	3.76 (1.34–10.58)		26 (5.95)	1.66 (0.82–3.33)	
7	1262 (44.14)	17 (1.35)	1.00		55 (4.36)	1.00	

CIND: cognitive impairment no dementia; PR: prevalence ratio, 95% CI: 95% confidence interval; * F Test, *p*-values in bold indicate statistical significance.

**Table 3:** Multivariate analysis of the association between adequate consumption of fruits, vegetables, and FV with cognitive impairment no dementia (CIND) in Brazilians aged 50 years or older, according to two adjustment models (*n* = 2865).

Fruits					
Model 1			Model 2		
	Adj PR (95% CI)	*p*-Value		Adj PR (95% CI)	*p*-Value
CIND	1.50 (1.26–1.80)	**<0.001**	CIND	1.47 (1.22–1.77)	**<0.001**
Age					
50 to 64	1.00				
65 to 74	1.15 (1.06–1.24)	**<0.001**			
75 or more	1.11 (0.98–1.25)	0.091			
Education					
Illiterate	1.00				
Primary	1.53 (1.03–2.27)	**0.036**			
Secondary/Higher	2.03 (1.35–3.05)	**0.001**			
**Vegetables**					
**Model 1**			**Model 2**		
CIND	0.19 (0.07–0.50)	**0.001**	CIND	0.19 (0.07–0.50)	**0.001**
Age			Hypertension		
50 to 64	1.00		No	1.00	**0.028**
65 to 74	1.16 (1.05–1.29)	**0.004**	Yes	1.15 (1.02–1.31)	
75 or more	1.23 (1.06–1.42)	**0.007**	Depression		
Skin colour			No	1.20 (1.03–1.40)	**0.021**
White	1.19 (1.01–1.41)	**0.045**	Yes	1.00	
Brown	1.00				
Black and others ^∞^	1.28 (1.05–1.58)	**0.017**			
Education					
Illiterate	1.00				
Primary	1.70 (1.20–2.41)	**0.003**			
Secondary/Higher	2.41 (1.66–3.50)	**<0.001**			
**FV**					
**Model 1**			**Model 2**		
CIND	0.20 (0.07–0.57)	**0.003**	CIND	0.20 (0.07–1.58)	**0.003**
Sex			DM		
Female	1.24 (1.08–1.41)	**0.002**	No	1.00	
Male	1.00		Yes	1.18 (1.01–1.38)	**0.043**
Age			Cerebrovascular Diseases		
50 to 64	1.67 (1.11–1.43)	**<0.001**	No	1.00	
65 to 74	1.34 (1.12–1.6)	**0.001**	Yes	1.39 (1.03–1.88)	**0.032**
75 and older	1.00				
Per capita income					
1º quartile	1.00				
2º quartile	1.21 (1.01–1.47)	**0.048**			
3º quartile	1.07 (0.88–1.30)	0.461			
4º quartile	1.16 (0.98–1.39)	0.088			
Education					
Illiterate	1.00				
Primary	1.64 (0.99– 2.66)	0.053			
Secondary/Higher	2.72 (1.64–4.51)	**0.000**			

Adj: adjustment, DM: diabetes mellitus, CIND: cognitive impairment no dementia; FV: fruits and vegetables, ^∞^ skin colour variable: black (*n* = 271) + yellow/indigenous (*n* = 19) Model 1: sex, age, skin colour, per capita income, education Model 2: Model 1 + smoking, sedentarism, obesity, DM, hypertension, depression, heart and cerebrovascular diseases, *p*-values in bold indicate statistical significance.

**Table 4:** Multivariate analysis of the association between adequate consumption of fruits, vegetables, and FV with dementias in Brazilians aged 50 years or older, based on two adjustment models (*n* = 2865).

Fruits					
Model 1			Model 2		
	Adj PR (95% CI)	*p*-Value		Adj PR (95% CI)	*p*-Value
Dementias	0.97 (0.71–1.33)	0.845	Dementias	1.06 (0.76–1.49)	0.698
Sex					
Female	1.09 (1.01–1.19)	**0.027**			
Male	1.00				
Age					
50 to 64	1.00				
65 to 74	1.15 (1.06–1.24)	**<0.001**			
75 or more	1.11 (0.98–1.26)	0.089			
Education					
Illiterate	1.00				
Primary	1.54 (1.03–2.32)	**0.038**			
Secondary/Higher	1.96 (1.26–3.03)	**0.003**			
**Vegetables**					
**Model 1**			**Model 2**		
Dementias	1.08 (0.77–1.50)	0.656	Dementias	0.77 (0.43–1.37)	0.370
Age			Hypertension		
50 to 64	1.00		No	1.00	
65 to 74	1.16 (1.05–1.29)	**0.005**	Yes	1.16 (1.01–1.33)	**0.034**
75 or more	1.22 (1.04–1.44)	**0.015**	DM		
Skin colour			No	1.00	
White	1.15 (0.94–1.40)	0.170	Yes	1.15 (1.02–1.31)	**0.027**
Brown	1.00				
Black and others ^∞^	1.30 (1.05–1.61)	**0.015**			
Education					
Illiterate	1.00				
Primary	1.71 (1.20–2.43)	**0.003**			
Secondary/Higher	2.57 (1.75–3.79)	**<0.001**			
**FV**					
**Model 1**			**Model 2**		
Dementias	1.05 (0.69–1.60)	0.808	Dementias	1.01 (0.67–1.51)	0.995
Sex			Obesity	-	0.108
Female	1.22 (1.06–1.39)	**0.005**	No	1.00	
Male	1.00		Yes	1.23 (1.02–1.47)	**0.027**
Age					
50 to 64	1.27 (1.12–1.65)	**<0.001**			
65 to 74	1.35 (1.00–1.45)	**0.003**			
75 or more	1.00				
Education					
Illiterate	1.00				
Primary	1.64 (1.01–2.66)	**0.043**			
Secondary/Higher	2.93 (1.77–4.85)	**<0.001**			

Adj: adjustment, DM: diabetes mellitus, FV: fruits and vegetables, ^∞^ skin colour variable: black (*n* = 271) + yellow/indigenous (*n* = 19). Model 1: sex, age, skin colour, per capita income, education Model 2: Model 1 + smoking, sedentarism, obesity, DM, hypertension, depression symptoms, dyslipidaemia, heart and cerebrovascular diseases, *p*-values in bold indicate statistical significance.

## Data Availability

The data used in this study are publicly available from the Brazilian Longitudinal Study of Aging (ELSI-Brazil). Researchers can access the dataset upon registration and approval through the official ELSI-Brazil website https://elsi.cpqrr.fiocruz.br (accessed on 25 May 2025). The study is conducted in accordance with national and international ethical standards, and all data are anonymized to ensure participant confidentiality. The data presented in this study are available on request from the corresponding author due to privacy.
